# Genetic diversity and evolutionary history of the *Schizothorax* species complex in the Lancang River (upper Mekong)

**DOI:** 10.1002/ece3.2319

**Published:** 2016-07-22

**Authors:** Weitao Chen, Yanjun Shen, Xiaoni Gan, Xuzhen Wang, Shunping He

**Affiliations:** ^1^ The Key Laboratory of Aquatic Biodiversity and Conservation of Chinese Academy of SciencesInstitute of HydrobiologyChinese Academy of SciencesWuhanHubei430072China; ^2^ Graduate School of Chinese Academy of SciencesBeijing10001China

**Keywords:** Divergence, genetic diversity, Lancang River, Pleistocene climate fluctuations, *Schizothorax* species complex

## Abstract

The genus *Schizothorax* (Cyprinidae), one of the most diverse genera of ichthyofauna of the Qinghai‐Tibetan Plateau (QTP), is a good candidate for investigating patterns of genetic variation and evolutionary mechanisms. In this study, sequences from the mitochondrial control region, the cytochrome *b* gene, and two nuclear genes were used to re‐examine the genetic diversity and investigate the evolutionary history of the *Schizothorax* species complex inhabiting the Lancang River. Three maternal clades were detected in the *Schizothorax* species complex, but frequent nuclear allele sharing also occurred among the three maternal clades. A discrepancy between topologies of mitochondrial and nuclear loci might result from introgression or/and incomplete lineage sorting. The divergence of the clades of the *Schizothorax* species complex was closely related to the Late Pliocene and Early Pleistocene orogenesis of the QTP and Southwest Mountains of China. Demographic analyses indicated that the species complex subsequently persisted in situ with stable populations during Pleistocene glacial cycling, which suggested that Pleistocene climate changes did not exert a remarkable influence on the species complex. Our study provides a comprehensive analysis of the genetic diversity and evolutionary history of the *Schizothorax* species complex in the Lancang River.

## Introduction

The Qinghai‐Tibetan Plateau (QTP) and China's Southwest Mountainous Region have undergone complex geological movements and climatic changes as the Late Miocene, resulting in a large number of new species, including plateau fish species (Cao et al. 1981; Shi et al. 1998; Liu et al. 1999; He et al. 2001, 2004; He and Chen 2006, 2007). The episodic geological changes in the QTP and China's Southwest Mountainous Region have rearranged the river drainage system of the plateau and its surrounding areas, possibly causing aquatic populations to become isolated evolve independently, and even form new species (Xiao et al. 2001; He and Chen 2006; Zhang et al. 2010; Yang et al. 2012). Southwestern China experienced drastic changes in its paleodrainage patterns. For example, during the Pliocene the upper reaches of the Yangtze River, Lancang River, and Nujiang River formed a tributary of the paleo‐Red River, which drained southwards into the South China Sea; these rivers were subsequently isolated owing to complex tectonic events. These historical breaks produced the high levels of genetic divergence among populations of fishes (Perdices et al. 2004; Guo et al. 2005; Yang et al. 2012).

Pleistocene glacial cycling frequently resulted in the periodic expansions and contractions of the population sizes and distribution ranges of species, but the effects on demographic history appear to vary among species as a function of cold tolerance and species distribution (Hewitt 1996, 2000). Compared with the heavy ice known to have covered high‐latitude continental Europe and North America, glaciations occurred discontinuously in the high mountains of the eastern edge of the QTP and are thought to have been absent in low‐elevation habitats (Shi et al. 1998; Zhou et al. 2006). Climatic perturbations appear to have mitigated demographic stresses for species at lower elevations compared with those at higher elevations (Qu et al. 2010; Lu et al. 2012).

Many large rivers (e.g., the Yangtze, Yellow River, Salween, and Mekong Rivers) flowing on the plateau provide abundant habitats for fishes (Wu 1992; Chen 1998, 2013). The Lancang River (upper Mekong), which originates from the QTP and flows into the Southwest Mountainous Region of China, has undergone complex river capture and reversal events as the Late Cenozoic (Clark et al. 2004). These historical drainage rearrangements have been revealed as the main driving force for shaping the current genetic structure of some species (Zhang et al. 2010, 2011), and even for facilitating speciation (He et al. 2001; He and Chen 2006).

**Figure 1:**
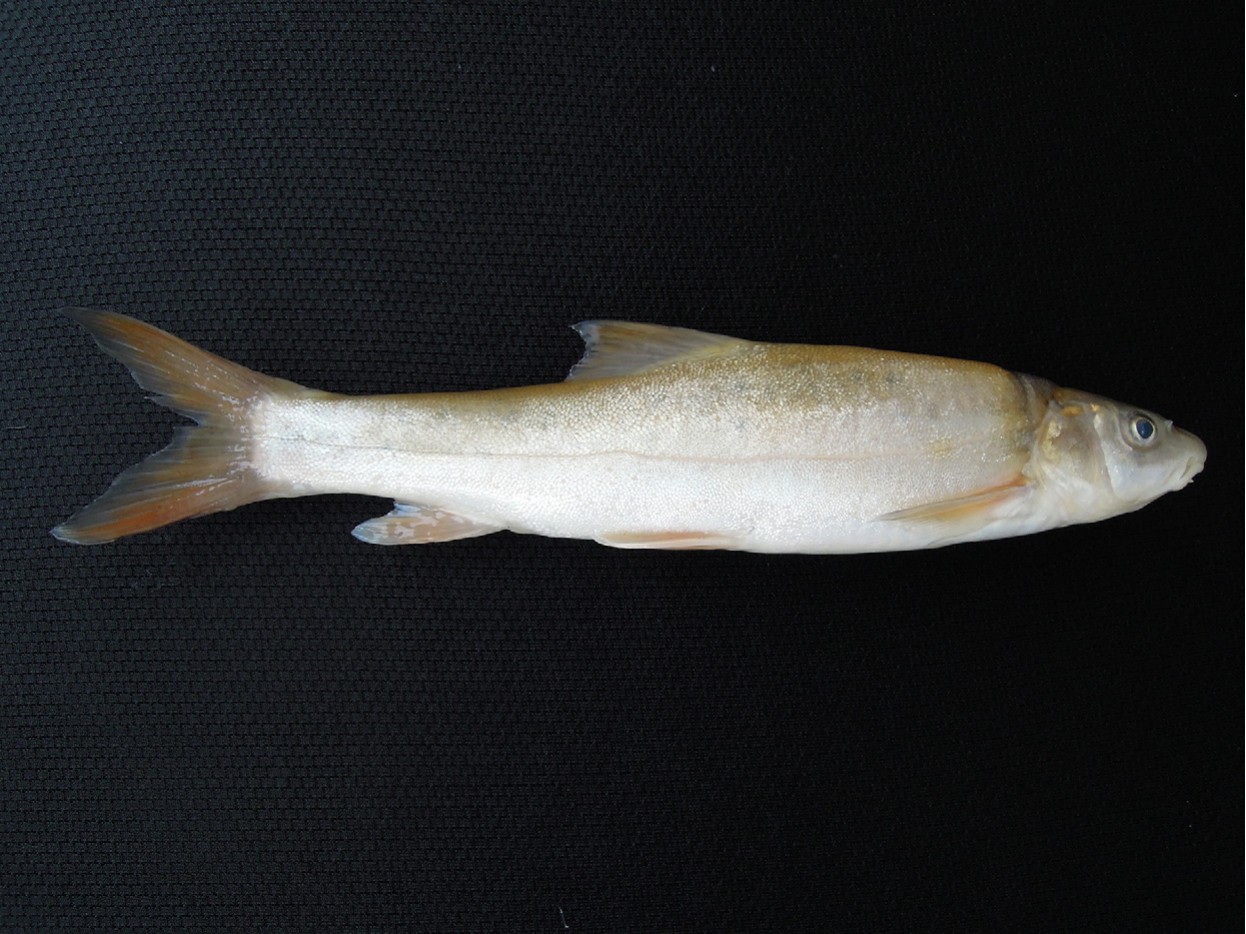
A representative organism of the *Schizothorax* species in the Lancang River.

The schizothoracine (Cyprinidae) fishes, representing the largest and most diverse taxon of the QTP ichthyofauna, exhibit exquisite adaptations to the rigorous conditions of the plateau (Wu and Wu 1992; Thompson et al. 2000; Bickler and Buck 2007). These fishes extensively dominate the torrential mountain streams and plateau lakes of the QTP, and the peripheral regions of the QTP (Cao et al. 1981; Chen and Cao 2000). The genus *Schizothorax* is one of the most diversified of the schizothoracines, comprising numerous species, and subspecies (Chen and Cao 2000). Five closely related *Schizothorax* species or subspecies (*S. lantsangensis*,* S. lissolabiatus*,* S. nudiventris*,* S. yunnanensis taliensis* and *S. yunnanensis yunnanensis*; Fig. 1) have been reported in the Lancang River basin. Compared with the broad distribution of *S. lissolabiatus* and *S. y. yunnanensis*,* S. lantsangensis,* and *S. nudiventris* only inhabit the main stream (Chen and Cao 2000; Chen 2013). Owing to human activities, *S. yunnanensis taliensis* has been reported to be near extinction and is rarely recorded in Erhai Lake (Chen 2013). Furthermore, *Schizothorax dolichonema* was previously reported to occur only in the Jinsha River (Upper Yangtze River), but this species has recently been noted in the upper Lancang River (Chen 2013). However, *S. dolichonema* is phylogenetically more closely related to the Jinsha River species complex than to the Lancang River species complex (Qi et al. 2012). The *Schizothorax* species complex in the Lancang River is an informative candidate for the study of the effects of the complex geological movements and Pleistocene glacial cycles on fish species on the QTP and in China's Southwest Mountainous Region.

The genetic diversity of the *Schizothorax* complex in the Lancang River is of great importance to the understanding of species diversity and establishment of a conservation strategy. However, the taxonomic status of the *Schizothorax* has long been a controversial issue (Cao et al. 1981; Wu and Wu 1992). Discrepancies between molecular and traditional morphological results have frequently occurred in this genus (He and Chen 2006; Yang et al. 2012). For example, the same morphological species from different drainages did not cluster together and different morphological species from the same drainage grouped together (He and Chen 2006; Yang et al. 2012). Even, species living in the same river drainage with a large distribution range may exhibit morphological variations, and the shape of the mouth and lips (important characteristics used for fishes' diets and species identification) can vary depending on the developmental stages of individuals, which may result in a lack of consensus regarding their taxonomy and lead to misidentification (Chen et al. 1982, Chen and Cao 2000). Thus, the species status based on morphotypes was questionable. More importantly, the DNA‐based studies performed to date on the *Schizothorax* species complex have only employed mitochondrial DNA (mtDNA) markers and have included relatively limited sample sizes and small geographic distributions (He and Chen 2006; Yang et al. 2012). A combined approach including both mtDNA and nuclear genes (nDNA) and larger sample sizes may offer better insights into the genetic diversity and evolutionary history of the species complex.

In the present study, we used both mtDNA and nDNA markers to investigate the genetic diversity and evolutionary history of the *Schizothorax* species complex in the Lancang River and to discuss the effects of the complex geological movements and climatic change on the species complex. Larger sample sizes were employed in our analyses than in previous study. Our main objectives were (1) to explore the genetic diversity of the *Schizothorax* species complex in the Lancang River; and (2) to investigate whether complex geological movements and Pleistocene climatic shifts have shaped the evolutionary history of this species complex.

## Methods and Materials

### Sample collection, laboratory techniques and molecular data

**Figure 2:**
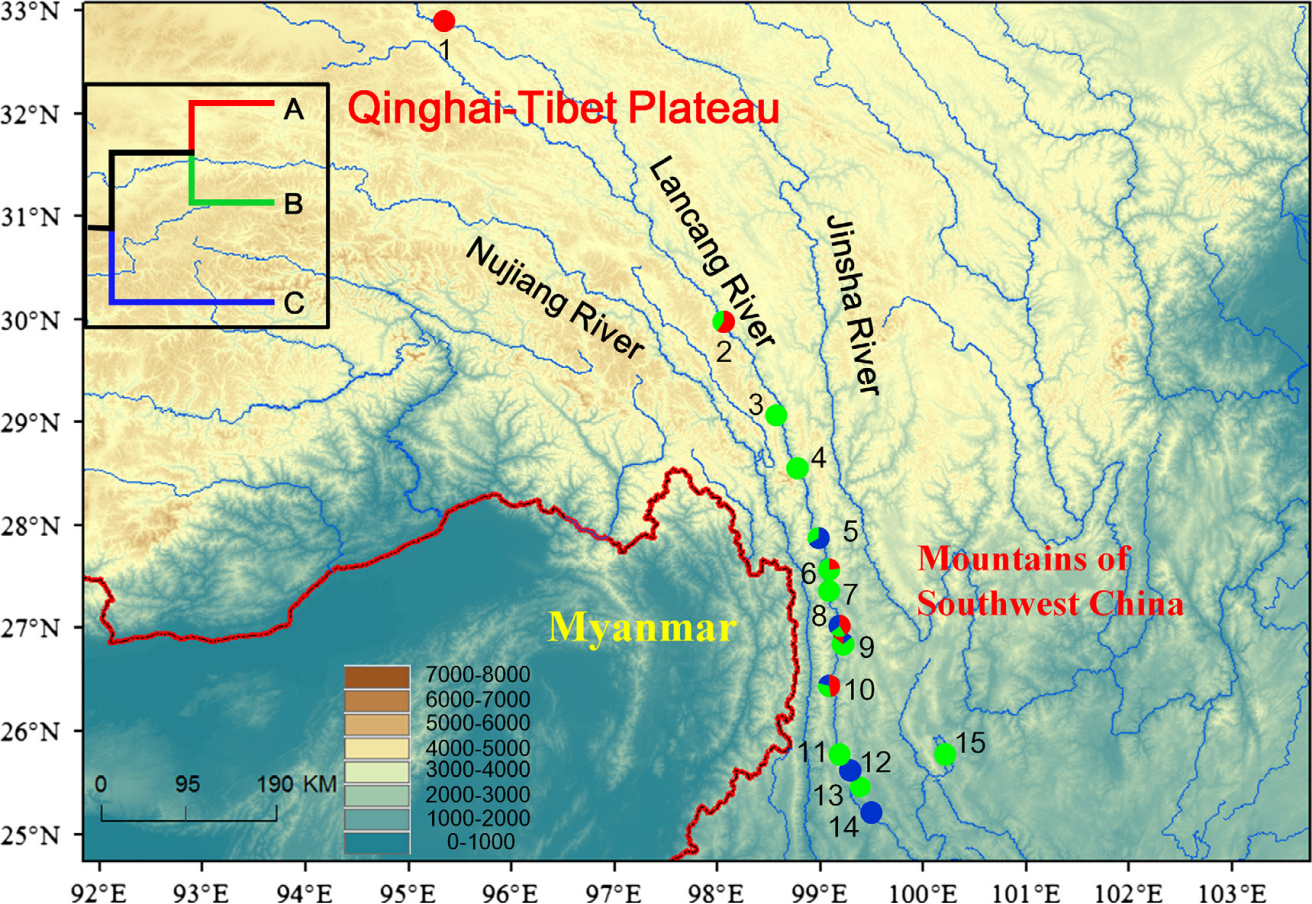
Map of the sampling sites for the *Schizothorax* species complex in the Lancang River. The site numbers are presented in Table S1. Populations are presented as pie diagrams with slice sizes proportional to the frequency of the major clades. The inset in the upper left corner shows the simplified maternal genealogy with major clades A, B and C. The colors of the pie diagrams and tree correspond to the clades in Figure 3A.

A total of 79 specimens of three morphologically distinct *Schizothorax* fishes (*S. lantsangensis*,* S. lissolabiatus* and *S. y. yunnanensis*) were collected from eight locations in the Lancang River basin from 2011 to 2013 (Table 1, Fig. 2 and Table S1). In addition, 22 published sequences belonging to five species (*S. dolichonema*,* S. lantsangensis*,* S. lissolabiatus*,* S. nudiventris* and *S. y. yunnanensis*) in the Lancang River basin from seven other locations were included in our study (Table 1, Fig. 2 and Table S1). A small piece of white muscle tissue or fin was dissected from the right side of each specimen. All tissue for genomic DNA extraction was preserved in 95% ethanol. Voucher specimens were deposited in the collection of the Freshwater Fish Museum of the Institute of Hydrobiology, Chinese Academy of Sciences.

**Table 1:** Sample locality number and sample sizes used in this study for each species from the Lancang River

Locality no.	*Schizothorax dolichonema*	*Schizothorax lissolabiatus*	*Schizothorax lantsangensis*	*Schizothorax nudiventris*	*Schizothorax yunnanensis yunnanensis*
1	*1*		*2*		
2		*1*	*3*		
3		*2*		*2*	
4		11			
5		*3*			
6		9	3		1
7		4			
8		6	7		4
9		6	1		
10		5	9		5
11				*2*	
12		*2 *+* *2			
13		*1*		*1*	*1*
14		*1*			
15		3			3
Total	1	56	25	5	14

Numbers in italics indicate sequences downloaded from the GenBank. More details are provided in Table S1.

The total genomic DNA was extracted from muscle tissue or fin tissue using standard salt extraction. The partial mitochondrial cytochrome *b* gene (*Cytb*) and control region (*CR*) were amplified for all individuals using the universal primers L14724 and H15915 (Xiao et al. 2001) and GEDL200 and GEDH860 (Zhao et al. 2009), respectively. Fragments of the recombinase‐activating gene 1 and gene 2 (*RAG‐1* and *RAG‐2*) were obtained from a subset of samples (58 individuals for *RAG‐1* and 68 individuals for *RAG‐2*). Primer information is listed in Table S2. The amplification of genomic DNA was conducted in a 30‐μL volume reaction with an initial denaturation period of 3–5 min at 94°C followed by 30–35 cycles of 94°C for 0.5–1 min, primer‐specific annealing temperature of 53–64°C (Additional file 2: Table S2) for 0.5–1 min, 72°C for 1–1.5 min and a single final extension at 72°C for 5–10 min. The amplified fragments were purified by 1.0% low‐melting agarose gel electrophoresis and sequenced with the identical primer pair using an ABI PRISM 3700 (Applied Biosystems, Foster City, CA) automatic DNA sequencer.

### Sequence analyses

The nucleotide sequences were initially edited using the DNASTAR multiple package (DNASTAR Inc., Madison, WI), aligned using the Muscle (Edgar 2004) and then optimized by eye in MEGA version 6.0 (Tamura et al. 2013). We combined the *Cytb* and *CR* sequences into the concatenated sequences (*CCR*) if individuals had the two gene fragments in the subsequent analyses. Although the schizothoracine fishes are polyploid (Yang et al. 2015), a single peak in the two nDNA gene sequence diagrams shows that the two genes are single copy genes. Nuclear gene sequences containing more than one ambiguous site were resolved using PHASE 2.1.1 (Stephens et al. 2001; Smith et al. 2005), for which input files were prepared using SEQPHASE (Flot 2010). Recombination tests for detecting the longest nonrecombining region for each locus were conducted using IMGC (Woerner et al. 2007).

### Phylogenetic and population analyses

First, given the close phylogenetic relationship between the *Schizothorax* species complex in the Lancang River and Salween River and the relatively small sample size in the previous phylogenetic analyses (He and Chen 2006; Yang et al. 2012), we added 447 *Cytb* sequences (unpublished data) of the *Schizothorax* species complex in the Salween River to confirm the monophyly of the *Schizothorax* species complex in the Lancang River. According to the phylogenetic analyses by He and Chen (2006) and Yang et al. (2012), the *Schizothorax* species complex from the same drainage clustered together and the same morphological species from different river system did not formed monophyletic groups. Therefore, other *Schizothorax* species complexes from the Jinsha River (Upper Yangtze River), Red River, Irrawaddy drainage and two *Gymnocypris* fishes (*G. eckloni* and *G. przewalskii*) downloaded from GenBank were used as the out‐group (Table S1). Because neighbor‐joining (NJ) analyses based on distance models performed better than Bayesian inference (BI) and maximum‐likelihood (ML) analyses, only the NJ tree was constructed using the Kimura 2‐parameter (K2P; Kimura 1980) model. The NJ tree was implemented in MEGA 6 (Tamura et al. 2013) using 1000 bootstrap replicates to assess the branch support. Furthermore, phylogenetic analyses of the *Schizothorax* species complex in the Lancang River of the *Cytb* data alone, and of the concatenated mtDNA sequences (*CCR*) were reconstructed used BI and ML analyses. The same outgroups using in the NJ analyses were applied in these analyses. The phylogenetic congruence of the *Cytb* and *CCR* data sets was examined in PAUP * v4b10 (Swofford 2002). For *CCR* sequences, *Schizothorax* species from the Tarim River basin, Tsangpo River and India downloaded from GenBank were used as the out‐group. Nucleotide substitution models were selected using the Akaike information criterion in MRMODELTEST version 2.3 (Nylander 2004). The best‐fit model was GTR + I + G for both *Cytb* and *CR*. BI analyses were performed using MRBAYES 3.1.2 (Ronquist and Huelsenbeck 2003). Three independent runs were performed for the two datasets for 20 million generations. Trees were sampled every 1000th generation, resulting in 20,000 trees, and the first 25% were discarded as burn‐in. For the *CCR* dataset, the BI analyses were partitioned by *Cytb* and *CR*. We checked for stationarity by ensuring that the potential scale reduction factor equaled 1, and that the average standard deviation of split frequencies between independent runs approached 0. We examined the MCMC samples in Tracer 1.5 (Rambaut and Drummond 2007) to ensure that all chains were sampled from the same target distribution. ML analyses were implemented in RAXML‐VI‐HPC (Stamatakis 2006) using the GTR + I + G model for the two datasets. Nodal support values were estimated from 1000 nonparametric bootstrap replicates.

We used NETWORK 4.6 (Bandelt et al. 1999) to construct a median‐joining network for the *Cytb*,* CR*,* RAG‐1,* and *RAG‐2*, separately. For mtDNA, we analyzed the *Cytb* and *CR* datasets directly. For the two nuclear genes, we used the longest nonrecombining region generated from IMGC. Genetic variation, including haplotype diversity (*h*) and nucleotide diversity (*π*) (Nei 1987) with standard errors, was calculated using DNASP 5.10 (Librado and Rozas 2009). Pairwise genetic differentiation (*F*_ST_) was calculated in ARLEQUIN 3.5 (Excoffier and Lischer 2010). Divergence among the clades was estimated using the K2P model as implemented in MEGA version 6.0.

### Historical demographic changes

We assessed the changes in demographic history using three methods. Only *Cytb* sequences were employed for demographic analyses. Because population subdivision may have masked the effect of expansion, we performed these analyses for each lineage separately. First, Ramos‐Onsins and Rozas's *R* ^2^ (*R* ^2^; Ramos‐Onsins and Rozas 2002) was calculated to find evidence of demographic expansions. The behavior of the *R* ^2^ test is superior for smaller sample sizes (Ramos‐Onsins and Rozas 2002). The significance of *R* ^2^ values was evaluated by comparing the observed value with a null distribution generated by 1000 replicates, using the empirical population sample size and observed number of segregating sites implemented in DNASP 5.10 (Librado and Rozas 2009). Mismatch distributions (Rogers and Harpending 1992) were used to infer the demographic history and were performed in ARLEQUIN 3.5 and DNASP 5.10. Bayesian skyline plots (BSPs; Drummond et al. 2005) were implemented in BEAST 1.6.1 (Drummond and Rambaut 2007), with 200 million generations to estimate the past historical effective population size for each clade. The best‐fit (Table S2) and the strict clock models were employed for each lineage. Four independent runs were performed and the resulting tree and log files were combined with LogCombiner v.1.6.1 (Rambaut and Drummond 2007). A divergence rate of 2.04% (calibrated in our analyses) per million years was applied in this study. The effective sample sizes (ESSs) were used for determining the Bayesian statistical significance of each parameter in TRACER 1.5.

### Gene flow

Potential gene flow among major lineages was estimated using the isolation with migration (IM) model with the program IMa2 (Hey 2010). We used two mtDNA gene fragments and the longest nonrecombining regions of the two nuclear loci for the IM analyses. The gene tree based on mtDNA markers was used as the guide tree. The method estimates the density functions and posterior‐probability densities of the IM model parameters using a Markov chain (MCMC) method (Hey and Nielsen 2007). The functions of the model parameters were first estimated in M‐mode with one million generations, and the first 10% were discarded as burn‐in. The MCMC run was repeated three times to confirm convergence. Using these functions, the marginal posterior distribution and the ML estimates of the demographic parameters were then estimated in the L‐mode. The HKY model of the DNA substitution was employed for both mtDNA and nDNA markers and 40 heated metropolis‐coupled Markov chains were employed to assure convergence. ESS were used to check whether the convergence was satisfactory.

### Divergence time estimation

To date, the extant clades from their divergence time, we used only *Cytb* and employed the relaxed molecular clock method incorporated in BEAST 1.6.1. We used a relaxed uncorrelated lognormal clock model, in which the rate in each branch is independently drawn from a lognormal distribution, and a Yule process was employed for speciation events. Two constraints were imposed on the tree to evaluate the time of divergence. Three species of the genus *Barbus* (*Barbus sclateri*,* Barbus guiraonis* and *Barbus callensis*) that were separated by the opening of the Gibraltar Strait (5 Ma) (Zardoya and Doadrio 1999), were used to calibrate the divergence times. The other calibration point was the separation time of two *Gymnocypris* fishes (*G. eckloni* and *G. przewalskii*) that were separated by the Gonghe Movement (0.15 Ma; Li and Fang 1998). The analysis was performed using 200 million generations, sampling every 2000th tree (the first 25% were treated as burn‐in), and with the GTR + I + G model of nucleotide substitution inferred using MRMODELTEST version 2.3. The sampled trees were annotated in TreeAnnotator v1.6.1 (BEAST software) and visualized in Figtree v1.3.1 (http://tree.bio.ed.ac.uk/software/figtree). The ESSs were used for determining the Bayesian statistical significance of each parameter in TRACER 1.5.

## Results

### Sequence information

For the in‐group mtDNA genes, we used a total of 101 *Cytb* sequences (79 de novo sequences and 22 published sequences) and 79 novel *CR* sequences. The 101 *Cytb* (1063 bp) sequences contained 133 variable sites and 94 parsimony‐informative sites. The *CR* sequences (670 bp) for in‐group individuals, exhibited 86 variable sites and 78 parsimony‐informative sites. The partition homogeneity test suggested no significant conflict between *CR* and *Cytb* (*P* = 0.94). We also used *CCR* in a phylogenetic analysis. A total of 29 haplotypes were defined from *Cytb* and *CR* from all in‐group sequences, respectively.

We obtained partial sequences for the two nuclear genes from a subset of all samples (Table S1). The dataset included 58 sequences (1466 bp) from *RAG‐1* and 68 sequences (1226 bp) from *RAG‐2*. The 58 *RAG‐1* sequences harbored 13 variable sites and 11 parsimony‐informative sites, and the 68 *RAG‐2* sequences contained 11 variable sites and 7 parsimony‐informative sites.

### Genealogy and nuclear gene networks

**Figure 3:**
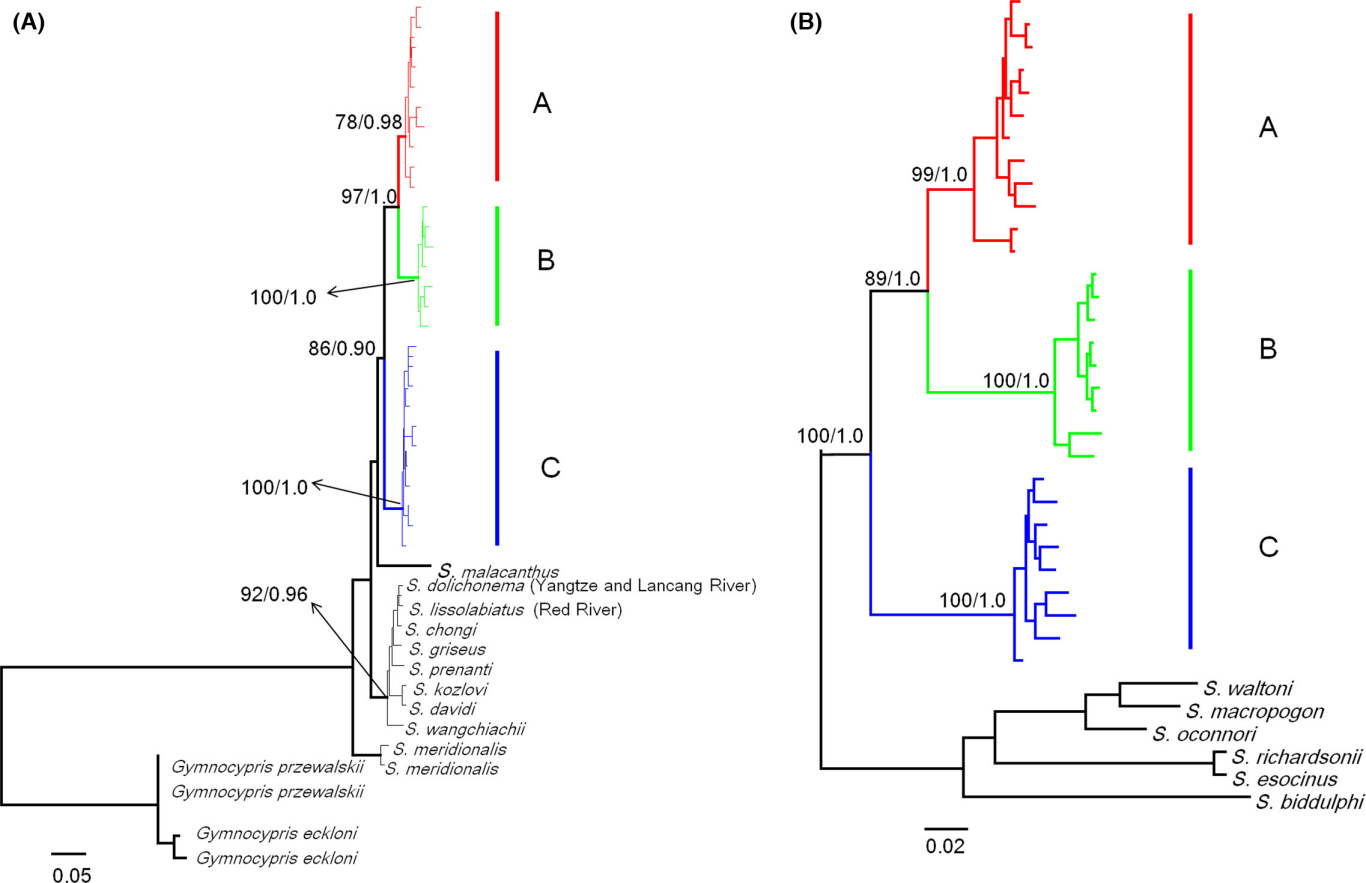
(A) Maternal genealogy from a maximum‐likelihood (ML) analysis for the *Schizothorax* species complex based on *Cytb* haplotypes. (B) Maternal genealogy from a ML analysis for the *Schizothorax* species complex based on *CCR* haplotypes. The numbers at the top are the bootstrap proportions from a ML analysis and Bayesian posterior probabilities.

The NJ trees (Fig. S1) showed that the species complexes in the Salween and Lancang Rivers were reciprocally monophyletic. The average standard deviation of split frequencies and the potential scale reduction factor of the BI analyses in our study approached 0.002 and 1.000, respectively. The phylogenetic trees of the in‐group obtained using *Cytb* via the BI and ML approaches showed a marked consistency in topological congruence, differing only in the support values for certain nodes; thus, only the ML tree is presented in Figure 3A. The species complex generated three highly supported clades (excluding *S. dolichonema*). Because *S. dolichonema* was only documented in the Jinsha River system and it has a close phylogenetic relationship to the species complex in the Jinsha River was close, we did not consider *S. dolichonema* in the subsequent analyses. Clade A consisted of specimens of *S. lantsangensis*, clade B contained three species (*S. lissolabiatus*,* S. nudiventris* and *S. y. yunnanensis*), and clade C consisted of *S. lissolabiatus,* and *S. y. yunnanensis*. The result for *CCR* was almost consistent with the *Cytb* trees, but it exhibited slightly different in support values for certain nodes (Fig. 3B).

*Cytb* and *CR* were used for constructing median‐joining networks. The grouping patterns of the median‐joining network for two mtDNA datasets resolved three clades and clearly showed the pattern of the shared haplotypes among *S. lissolabiatus*,* S. nudiventris*, and *S. y. yunnanensis* (Fig. S2). *S. lantsangensis* did not share haplotypes with other species (Fig. S2). Three morphologic species (*S. lissolabiatus*,* S. nudiventris,* and *S. y. yunnanensis*) shared *Cytb* haplotypes in the clade B, and two morphological species (*S. lissolabiatus* and *S. y. yunnanensis*) frequently shared *Cytb* and *CR* haplotypes in the clades B and C. The scenario of the mtDNA haplotype shared among these morphological species might be interpreted by incomplete lineage sorting, rapid radiation or misidentification (He and Chen 2006; Yang et al. 2012).

**Figure 4:**
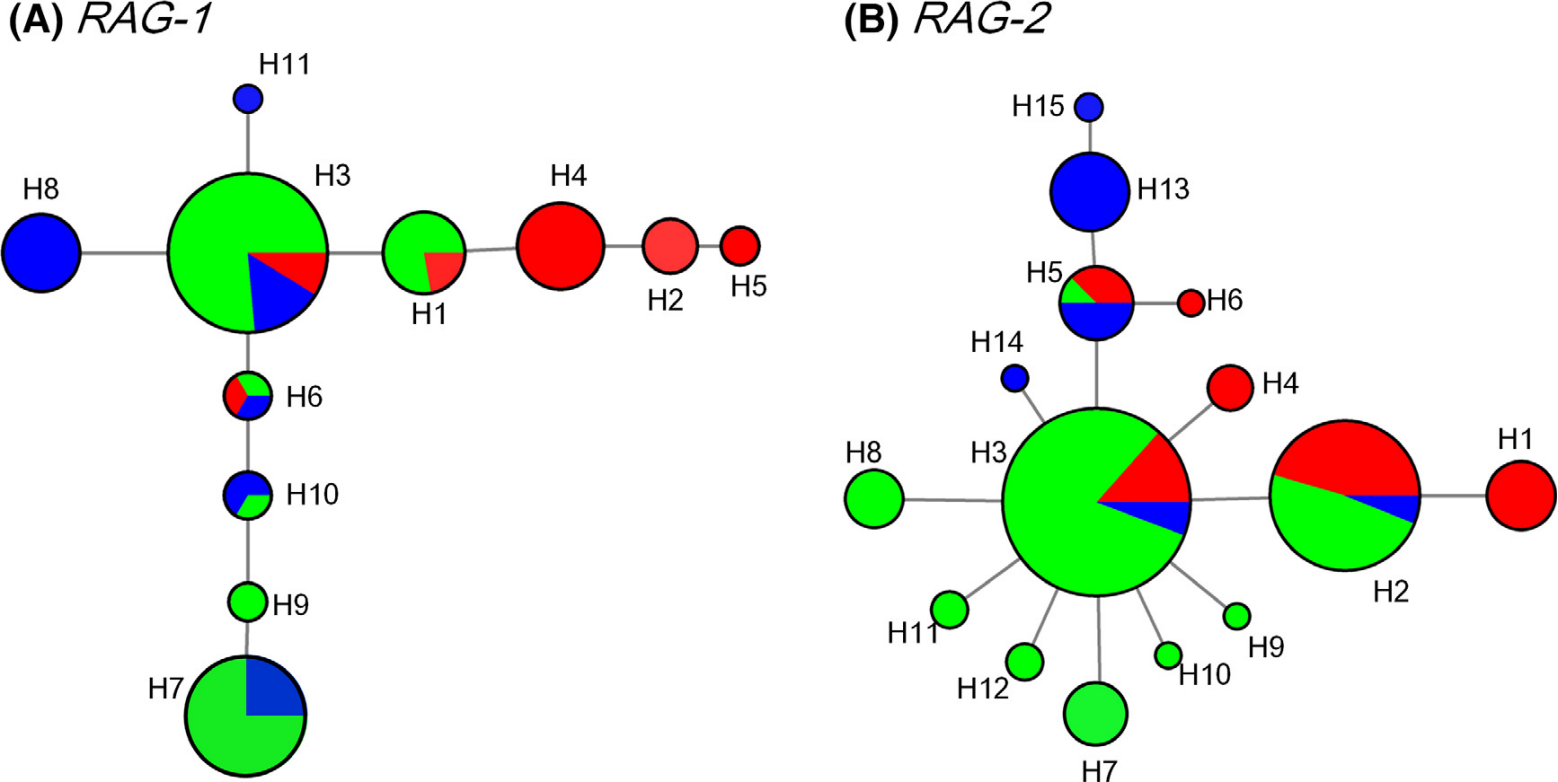
Median‐joining network of the nuclear gene fragments for the *Schizothorax* species complex. Each circle color represents different clades (red, A; green, B; and blue, C) and is scaled by its frequency in the entire sample. (A) *RAG‐1*. (B) *RAG‐2*.

Given the limited number of potentially parsimony‐informative sites, we built median‐joining networks using only the longest nonrecombining regions of the two nuclear genes (Fig. 4A and B). This strategy resulted in 11 haplotypes from *RAG‐1* and 15 haplotypes from *RAG‐2*. In the *RAG‐1* network, two haplotypes (H3 and H6) were shared by the three clades (Fig. 4A). In addition, haplotypes were often shared between the clades A and B, and between clades B and C. In the *RAG‐2* network, we detected that three haplotypes (H2, H3 and H5) that were shared by the three clades and no haplotypes that were shared by the two clades (Fig. 4B).

### Genetic diversity and demographic history

We calculated the haplotype diversity and the nucleotide diversity of the three mtDNA clades based on the gene trees (Table 2). Except for the *F*_ST_ value between clades B and C based on *RAG‐1* sequences, *F*_ST_ values were statistically significant ranging from 0.174 to 0.942 based on both mtDNA and nDNA datasets (Table 2). The mtDNA genetic divergence determined based on *Cytb* and *CCR*, ranged from 3.55 to 6.11% and 3.97 to 6.29%, respectively, between clades A–C (Table 3).

**Table 2:** Summary of genetic diversity and neutrality tests of all clades

Matriline	Locus	*N*	*N*_h_	*h* ± SD	*π *± SD	*R* ^2^	*P*
A	*Cytb*	25	10	0.890 ± 0.033	0.0060 ± 0.0007	0.12	0.564
	*CCR*	20	12	0.937 ± 0.033	0.0067 ± 0.0011	–	–
	*RAG‐1*	26	9	0.825 ± 0.060	0.0016 ± 0.0002	–	–
	*RAG‐2*	36	6	0.757 ± 0.048	0.0010 ± 0.0001	–	–
B	*Cytb*	57	7	0.528 ± 0.070	0.0034 ± 0.0008	0.10	0.093
	*CCR*	46	9	0.741 ± 0.059	0.0044 ± 0.0008	–	–
	*RAG‐1*	72	14	0.794 ± 0.032	0.0022 ± 0.0001	–	–
	*RAG‐2*	78	11	0.689 ± 0.047	0.0008 ± 0.0001	–	–
C	*Cytb*	18	11	0.948 ± 0.030	0.0031 ± 0.0004	0.14	0.232
	*CCR*	13	9	0.936 ± 0.051	0.0074 ± 0.0007	–	–
	*RAG‐1*	18	6	0.817 ± 0.058	0.0023 ± 0.0002	–	–
	*RAG‐2*	22	7	0.797 ± 0.067	0.0012 ± 0.0002	–	–

*h*, haplotype diversity; *π*, nucleotide diversity; SD, standard error; *N*, number of individuals; *N*_h_, number of haplotypes; *R* ^2^, Ramos‐Onsins and Rozas's *R* ^2^ test; and *P*, probability value.

**Table 3:** Pairwise *F*_ST_ values and genetic distances among the three clades

	*F*_ST_				K2P distance	
	*Cytb*	*CCR*	*RAG‐1*	*RAG‐2*	*Cytb*	*CCR*
A versus B	**0.876** (0.000)	**0.868** (0.000)	**0.350** (0.000)	**0.174** (0.000)	0.0355	0.0397
A versus C	**0.895** (0.000)	**0.846** (0.000)	**0.411** (0.000)	**0.410** (0.000)	0.0475	0.0488
B versus C	**0.942** (0.000)	**0.914** (0.000)	−0.011 (0.076)	**0.415** (0.000)	0.0611	0.0629

Values in the brackets are exact values of the significance test. The *F*_ST_ values in bold are significant (*P* < 0.05).

**Figure 5:**
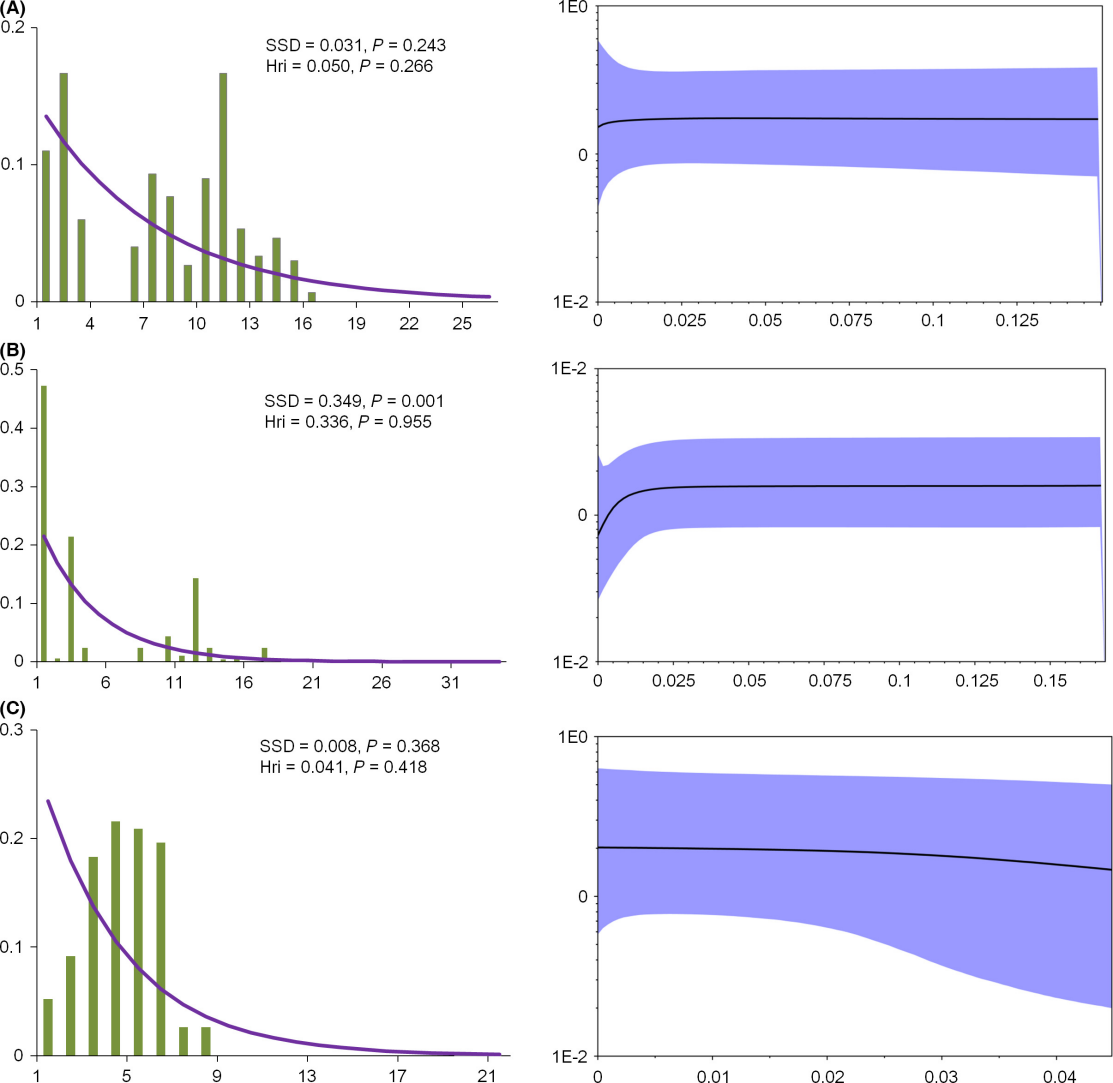
Mismatch distribution and Bayesian skyline plots (BSPs) analysis. (A) Clade A. (B) Clade B. (C) Clade C. Pictures on left are results of the mismatch distribution. The abscissa indicates the number of pairwise differences between compared sequences. The ordinate is the frequency for each value. The histograms are the observed frequencies of pairwise divergences among sequences and the line refers to the expectation under the model of population expansion. The pictures on the right are the results of BSP. The abscissa shows the time in millions of years ago (Ma). The ordinate shows the estimated effective population size. The estimates of means are joined by a solid line while the shaded range delineates the 95% HPD limits. SSD, sum of squared distribution; Hri, Harpending's raggedn ess index; *P*, probability value.

Neutrality tests of *R* ^2^ yielded no statistically significant values for all clades (Table 2). The sum of square deviations and raggedness index suggested that the curves did not significantly deviate from the distributions expected under a model of sudden demographic expansion (Fig 5). Bimodal or multimodal profiles were identified in clades A and B based on the mismatch analyses (Fig. 5). In addition, the BSPs suggested that the effective population size was relatively stable or slowly decreasing in all tested clades (Fig. 5).

### Gene flow

**Figure 6:**
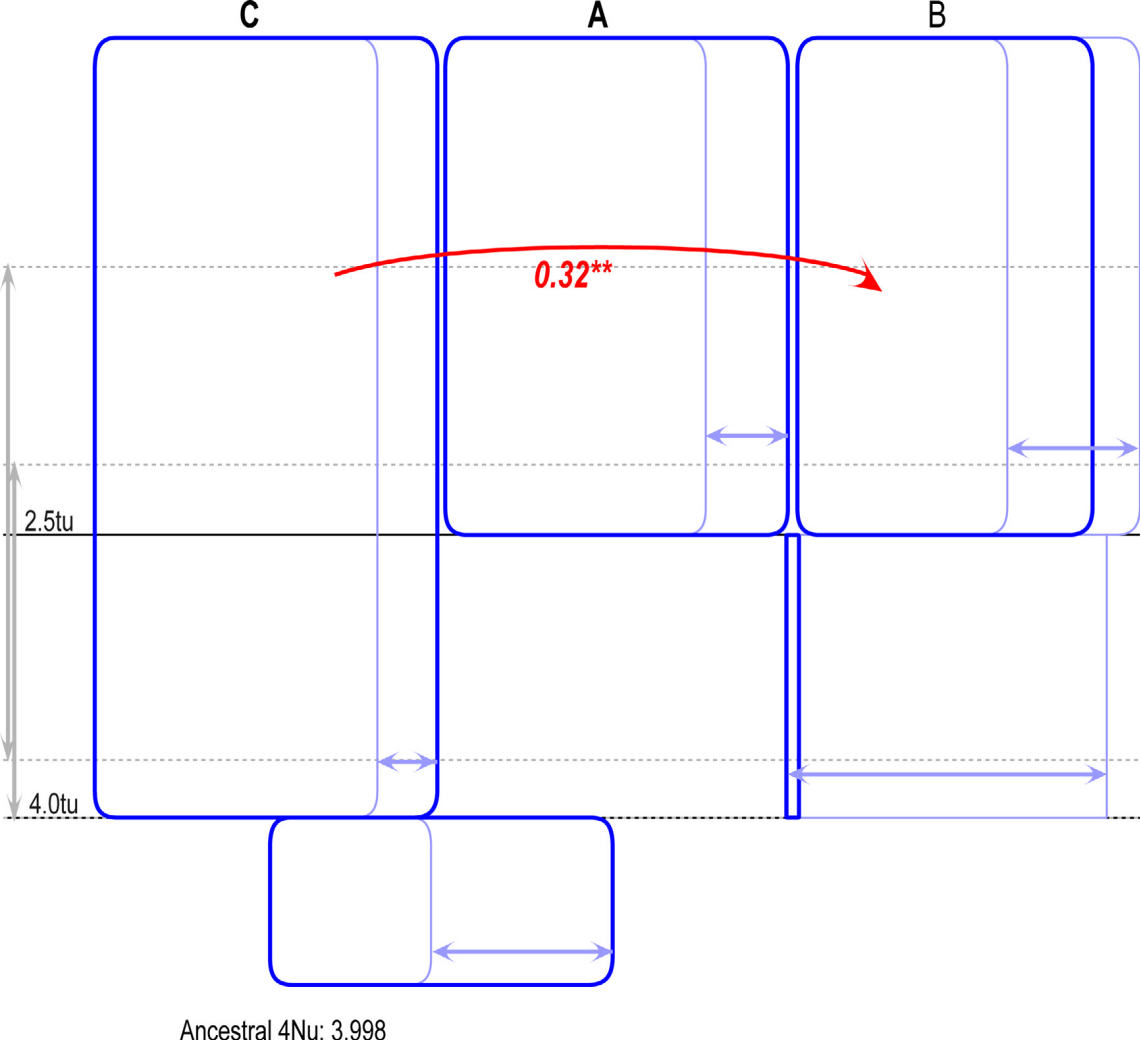
Isolation with migration analyses for the three clades of the *Schizothorax* species complex. The arrows represent migration directions from the source population to the receiving population; the numbers next to arrows are 2NM values. Only statistically significant cases of gene flow are presented. **P < 0.01.

Potential gene flow was examined among the three lineages (Fig. 6). We detected one statistically significant migration event from C to B (2NM = 0.32), whereas significant gene flow was not observed in the reverse direction. No significant gene flow was examined between clade A and the other two clades.

### Divergence time estimation

**Figure 7:**
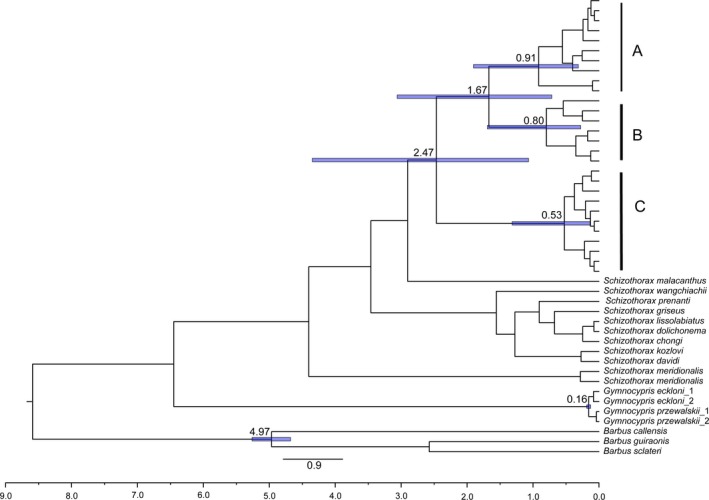
Divergence time estimates for the *Schizothorax* species complex. Branch lengths are proportional to divergence times (Ma). Tree topology is derived from BEAST. The bars on the nodes are 95% confidence intervals. Clades A, B, and C correspond to those in Figure 3.

The average divergence times of the entire in‐group are presented in Fig. 7. Clade C diverged at 2.47 Ma (95% highest posterior density [HPD], 1.07–4.35 Ma), and clades A and B diverged at 1.67 Ma (95% HPD, 0.72–3.06 Ma). In addition, within A, B, and C, subclades diverged between 0.52 and 0.94 Ma (95% HPD, 0.13–1.32 to 0.31–1.90 Ma).

## Discussion

### Genetic diversity and possible driver of lineage divergence

In our study, three highly supported clades (A–C) were detected based on mtDNA datasets. This finding was consistent with the results reported by Yang et al. (2012), but revealed one more clade than the study by He and Chen (2006). The monophyly of the *Schizothorax* species complex in the Lancang River was demostrated by the phylogenetic trees including the species complex in the Salween River (Fig. S1). Therefore, we can rule out the possibility of the colonization from another river system. In addition to high levels of genetic divergence among clades based on different datasets, we can conclude that the species complex in the Lancang River harbors high genetic diversity. Although deep divergence among the three clades was observed based on mtDNA datasets, we unexpectedly found that haplotype sharing frequently occurred among the three clades from the two nDNA networks. The discrepancy between topologies observed in mtDNA fragments and nDNA genes might be indicative of incomplete lineage sorting, or introgression, or both (McGuire et al. 2007). In our study, substantial gene flow was detected from clade C to clade B (2NM = 0.32), and the sympatric distribution probably reflects the ongoing gene flow among the clades. However, the detected levels of gene flow might not be high enough to prevent divergence; a 2NM greater than one would limit the divergence process in the absence of selection (Hey 2010). However, our results cannot rule out the effects of incomplete lineage sorting on the discordant patterns between mitochondrial and nuclear loci because the rate of nucleotide substitution of the nuclear protein‐coding genes appears to be roughly an order of magnitude slower than that of mtDNA fragments in many taxa (Simon et al. 2006). Additionally, in case of incomplete lineage sorting, alleles from a common ancestor that are retained in the descendant species are expected to be randomly distributed in the descendant populations.

The orogenesis of the QTP is considered to be responsible for driving speciation in many taxa through vicariance or changes in environmental patterns (Macey et al. 1998; He et al. 2001; Luo et al. 2004; Liu et al. 2006; Che et al. 2010; Lu et al. 2012; Zhou et al. 2012). The three clades (A–C) observed in the Lancang River exhibited high levels of divergence. The increased levels of divergence potentially reflect long‐term isolation, which is likely associated with the complex geological history of the QTP. The divergence of A, B, and C dates from 1.67 to 2.47 Ma based on *Cytb* sequences, which suggests that these clades have been separated as the beginning of the Late Pliocene (Cui et al. 1996; Li et al. 2001). This period is broadly consistent with the rapid and dramatic uplift of the QTP and adjacent Southwest China (3.6–0.15 Ma) (Zheng et al. 2000; Li et al. 2001). The plateau has experienced three phases of intense uplift as the beginning of the Late Pliocene: 3.6–1.7, 1.1–0.6, and 0.15 Ma (Li and Fang 1998; Shi et al. 1998; Li et al. 2001). These phases of geological movement are responsible for habitat fragmentation and the complex development of watersheds and watercourses, which could block or inhibit dispersion and gene flow between fish populations. Many fish species have exhibited similar effects in the plateau drainages (He et al. 2001, 2004; Peng et al. 2006; He and Chen 2007; Yang et al. 2012; Wu et al. 2013). The present pattern regarding the sympatric distribution of the three clades may be due to repeated river capture and reversal events associated with the geological events of the QTP and the Southwest Mountains as the late Pliocene (Clark et al. 2004). Thus, the complex geological history appears to be responsible for driving the formation of the current diversity pattern of the *Schizothorax* species complex in the Lancang River.

### Stabilization during Pleistocene climatic changes

Rapid population expansion during interglacial periods seems to have shaped the current patterns of genetic diversity (Hewitt 2000, 2004; Zhao et al. 2011; Lu et al. 2012; Zhou et al. 2013, 2014). However, in our study, demographical expansion seemed not to have occurred in the *Schizothorax* species complex in the Lancang River. The neutrality tests, mismatch distribution analyses and BSPs did not detect a signal of rapid population expansion for the three clades. Long‐term stability of populations also occurs in *Terminalia franchetii* (Zhang et al. 2011), *Poropuntius huangchuchieni* (Wu et al. 2013), *Quasipaa boulengeri* (Yan et al. 2013) and some birds (Qu et al. 2014) in Southwest China. Therefore, Pleistocene climatic fluctuations do not appear to be an important driver for some species in Southwest China.

Two major factors may explain the absence of historical expansion. First, Southwest China is a complex mosaic of several mountains that experienced a relatively stable climate (Weaver et al. 1998; Pinot et al. 1999). Furthermore, ice caps are considered to have been absent in this region because its elevation has remained below the snow line. The snow line in many mountain ranges during both the Last Glacial Maximum (0.025–0.018 Ma) and the extensive glacial period (0.5–0.175 Ma) exceeded approximately 3300 m (Shi et al. 1997; Liu et al. 1999). Second, the aquatic habitat provides relatively stable conditions today and probably also during Pleistocene climatic cycling. Thus, without pressure from a lack of suitable habitats during glaciations, the species complex might not have undergone drastic demographic fluctuations, such as bottlenecks and expansion. However, more data are needed to test these hypotheses in the future owing to limited sample sizes and molecular information in the current study.

## Conclusions

Our study assembles mtDNA and nDNA markers to infer genetic diversity and the evolutionary history of the *Schizothorax* species complex in the Lancang River. The results confirm that three clades are found in the Lancang River and support the hypothesis that geological events during the Late Pliocene and Early Pleistocene have driven the divergence of the *Schizothorax* species complex. However, a discrepancy between topologies in mtDNA fragments and nDNA genes was observed, which might result from introgression and/or incomplete lineage sorting. Surprisingly, Pleistocene climatic fluctuations do not play a key role in historical demography of the species complex. Because it is a completely aquatic genus, habitat destruction and water pollution due to economic and agricultural development (e.g., water construction and domestic wastewater), and overfishing for human consumption are the main threats to the *Schizothorax* (Dudgeon 2011). Owing to limited resources, it may be necessary to prioritize the protection of the remaining populations. Furthermore, protection measurements for the *Schizothorax* species complex should focus on habitat conservation.

## Conflict of Interest

The authors declare that they have no competing interests.

## Data Accessibility

DNA sequences have been deposited in GenBank under the accession nos. KT033915–KT033934, KT03395–KT033997, KT034070–KT034082, KT034092–KT034103, KT034120, KT034124, KT034128–KT034130, KT034132, KT034150–KT034152, KT034176–KT034188, KT034200, KT034202–KT034212, KT034214, KT034226–KT034228, KT034239–KT034258, KT034276–KT034321, KT034396–KT034405, KU612445–KU612477, and KU612479–KU612521. Details regarding individual samples are available in Table S1.

## Supporting information


**Figure S1.** Neighbor‐joining tree of the *Schizothorax* species complex in several drainages based on *Cytb* sequences.

**Figure S2.** (A) Median‐joining network of *Cytb* for the *Schizothorax* species complex. (B) Median‐joining network of *CR* for the *Schizothorax* species complex.

**Table S1.** Detailed information for specimens of the *Schizothorax* species complex from the Lancang River and outgroups included in this study.

**Table S2.** Primers used for PCR and sequencing.
