# Supplementary figures and images for: Genetic diversity and evolutionary history of the Schizothorax species complex in the Lancang River (upper Mekong)
Source: Ecol Evol. 2016 Jul 22;6(17):6023–36. doi: 10.1002/ece3.2319 (PMC5016629; doi:10.1002/ece3.2319)

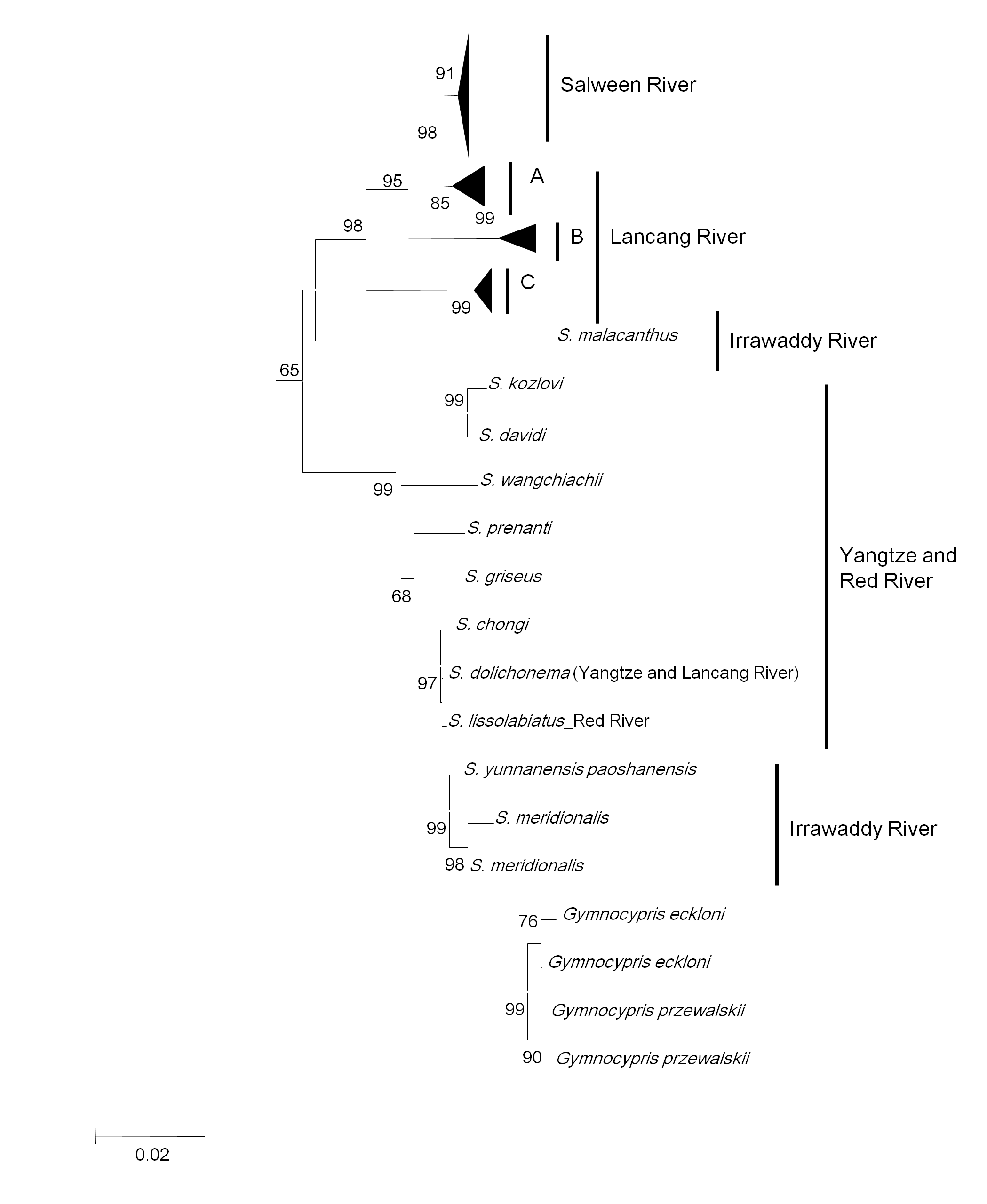

Supplement: Supplementary file 1 — Figure S1. Neighbor‐joining tree of the Schizothorax species complex in several drainages based on Cytb sequences. [file ECE3-6-6023-s001.tif]

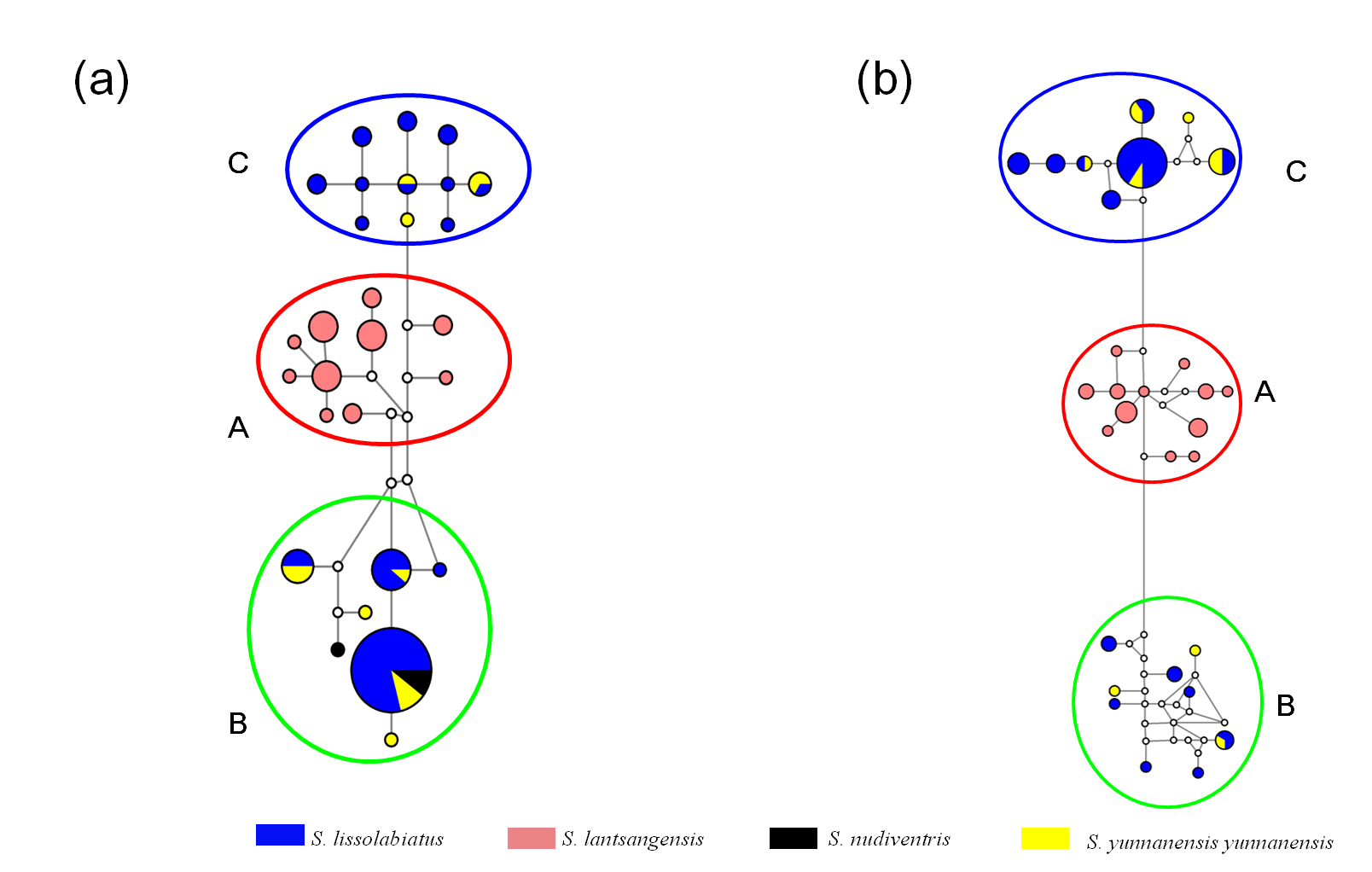

Supplement: Supplementary file 2 — Figure S2. (A) Median‐joining network of Cytb for the Schizothorax species complex. (B) Median‐joining network of CR for the Schizothorax species complex. [file ECE3-6-6023-s002.tif]
