# Supplementary material for: Genetic diversity and evolutionary history of the Schizothorax species complex in the Lancang River (upper Mekong)
Source: Ecol Evol. 2016 Jul 22;6(17):6023–36. doi: 10.1002/ece3.2319 (PMC5016629; doi:10.1002/ece3.2319)
Supplement: Supplementary file 3 — Table S1. Detailed information for specimens of the Schizothorax species complex from the Lancang River and outgroups included in this study. [file ECE3-6-6023-s003.doc]

**Table S1 Detailed information for specimens of** ***Schizothorax* species complex from the Lancang River and outgroups included in this study.**

| **Locality** | **Species name** | **Locality Name** | **Longitude** | **Latitude** | **Elevation** | **Sample** | **Number of** |  |  |  |  |
| --- | --- | --- | --- | --- | --- | --- | --- | --- | --- | --- | --- |
| **number** |  |  |  |  |  | **number** | **specimens** | *Cytb* | *CR* | *RAG-1* | *RAG-2* |
| Ingroup |  |  |  |  |  |  |  |  |  |  |  |
| 1 | *S. dolichonema* | Zadoi, Qinghai, China |  |  |  |  | 1 | JQ082344* |  |  |  |
| 1 | *S.lantsangensis* | Zadoi, Qinghai, China |  |  |  |  | 1 | DQ646882* |  |  |  |
| 1 | *S.lantsangensis* | Zadoi, Qinghai, China |  |  |  |  | 1 | JQ082343* |  |  |  |
| 2 | *S.lantsangensis* | Chuka, Tibet, China |  |  |  |  | 3 | DQ126126* |  |  |  |
| 2 | *S. lissolab1atus* | Chuka, Tibet, China |  |  |  |  | 2 | DQ126127* |  |  |  |
| 3 | *S. nudiventris* | Quzika, Mangkang, Tibet |  |  |  |  | 2 | EU158026* |  |  |  |
| 3 | *S. lissolab1atus* | Quzika, Mangkang, Tibet |  |  |  |  | 1 | EU158042* |  |  |  |
| 4 | *S. lissolab1atus* | Adong River, Deqin, Yunnan | 98.798 | 28.544 |  | ihb201306175 |  | KT034303 | KT033958 | KU612474 | KU612516 |
| 4 | *S. lissolab1atus* | Adong River, Deqin, Yunnan | 98.798 | 28.544 |  | ihb201306176 |  | KT034301 | KT033975 | KU612473 | KU612514 |
| 4 | *S. lissolab1atus* | Adong River, Deqin, Yunnan | 98.798 | 28.544 |  | ihb201306177 |  | KT034298 | KT033966 |  | KT034204 |
| 4 | *S. lissolab1atus* | Adong River, Deqin, Yunnan | 98.798 | 28.544 |  | ihb201306178 |  | KT034291 | KT033963 | KU612460 | KU612502 |
| 4 | *S. lissolab1atus* | Adong River, Deqin, Yunnan | 98.798 | 28.544 |  | ihb201306179 |  | KT034292 | KT033971 | KU612452 | KU612493 |
| 4 | *S. lissolab1atus* | Adong River, Deqin, Yunnan | 98.798 | 28.544 |  | ihb201306180 |  | KT034285 | KT033962 | KU612464 | KU612506 |
| 4 | *S. lissolab1atus* | Adong River, Deqin, Yunnan | 98.798 | 28.544 |  | ihb201306181 |  | KT034311 | KT033979 | KU612450 | KU612491 |
| 4 | *S. lissolab1atus* | Adong River, Deqin, Yunnan | 98.798 | 28.544 |  | ihb201306182 |  | KT034304 | KT033961 | KU612471 | KU612512 |
| 4 | *S. lissolab1atus* | Adong River, Deqin, Yunnan | 98.798 | 28.544 |  | ihb201306183 |  | KT034306 | KT033982 | KU612468 | KU612509 |
| 4 | *S. lissolab1atus* | Adong River, Deqin, Yunnan | 98.798 | 28.544 |  | ihb201306184 |  | KT034309 | KT033980 | KU612451 | KU612492 |
| 4 | *S. lissolab1atus* | Adong River, Deqin, Yunnan | 98.798 | 28.544 |  | ihb201306185 |  | KT034312 | KT033985 | KT034132 | KT034214 |
| 5 | *S. lissolab1atus* | Badi, Wixi, Yunnan |  |  |  |  | 1 | EU158043* |  |  |  |
| 5 | *S. lissolab1atus* | Badi, Wixi, Yunnan |  |  |  |  | 1 | EU158044* |  |  |  |
| 5 | *S. lissolab1atus* | Badi, Wixi, Yunnan |  |  |  |  | 1 | EU158045* |  |  |  |
| 6 | *S. lissolab1atus* | Biluo River, Wixi, Yunnan | 99.062 | 27.411 |  | ihb201306152 |  | KT034305 | KT033984 | KT034128 | KT034210 |
| 6 | *S. lissolab1atus* | Biluo River, Wixi, Yunnan | 99.062 | 27.411 |  | ihb201306153 |  | KT034299 | KT033960 | KU612476 | KU612518 |
| 6 | *S. lissolab1atus* | Biluo River, Wixi, Yunnan | 99.062 | 27.411 |  | ihb201306154 |  | KT034288 | KT033972 | KU612469 | KU612510 |
| 6 | *S. lissolab1atus* | Biluo River, Wixi, Yunnan | 99.062 | 27.411 |  | ihb201306155 |  | KT034295 | KT033974 | KU612462 | KU612504 |
| 6 | *S. lissolab1atus* | Biluo River, Wixi, Yunnan | 99.062 | 27.411 |  | ihb201306156 |  | KT034308 | KT033981 | KU612475 | KU612517 |
| 6 | *S. lissolab1atus* | Biluo River, Wixi, Yunnan | 99.062 | 27.411 |  | ihb201306157 |  | KT034279 | KT033957 | KU612466 | KU612507 |
| 6 | *S. lissolab1atus* | Biluo River, Wixi, Yunnan | 99.062 | 27.411 |  | ihb201306158 |  | KT034294 | KT033976 | KU612458 | KU612498 |
| 6 | *S. lissolab1atus* | Biluo River, Wixi, Yunnan | 99.062 | 27.411 |  | ihb201306161 |  | KT034287 | KT033977 |  | KU612515 |
| 6 | *S. lissolab1atus* | Biluo River, Wixi, Yunnan | 99.062 | 27.411 |  | ihb201306150 |  | KT034286 | KT033973 | KU612454 | KU612495 |
| 6 | *S.lantsangensis* | Biluo River, Wixi, Yunnan | 99.062 | 27.411 |  | ihb201306149 |  | KT034239 | KT033916 | KT034096 | KT034180 |
| 6 | *S.lantsangensis* | Biluo River, Wixi, Yunnan | 99.062 | 27.411 |  | ihb201306159 |  | KT034247 | KT033932 | KU612445 | KU612484 |
| 6 | *S.lantsangensis* | Biluo River, Wixi, Yunnan | 99.062 | 27.411 |  | ihb201306160 |  | KT034248 | KT033928 | KT034095 | KT034179 |
| 6 | *S. yunnanensis* | Biluo River, Wixi, Yunnan | 99.062 | 27.411 |  | ihb201306151 |  | KT034401 | KT034075 | KU612477 | KU612519 |
| 7 | *S. lissolab1atus* | Baixunji, Wixi, Yunnan | 99.090 | 27.349 |  | ihb201306141 |  | KT034300 | KT033968 | KU612465 |  |
| 7 | *S. lissolab1atus* | Baixunji, Wixi, Yunnan | 99.090 | 27.349 |  | ihb201306142 |  | KT034283 | KT033969 | KU612457 | KU612497 |
| 7 | *S. lissolab1atus* | Baixunji, Wixi, Yunnan | 99.090 | 27.349 |  | ihb201306144 |  | KT034297 | KT033965 | KU612461 | KU612503 |
| 7 | *S. lissolab1atus* | Baixunji, Wixi, Yunnan | 99.090 | 27.349 |  | ihb201306145 |  | KT034290 | KT033988 | KU612453 | KU612494 |
| 8 | *S. lissolab1atus* | Biyu River, Lanping, Yunnan | 99.153 | 26.980 |  | ihb201306132 |  | KT034319 | KT033991 | KT034129 | KT034211 |
| 8 | *S. lissolab1atus* | Biyu River, Lanping, Yunnan | 99.153 | 26.980 |  | ihb201306133 |  | KT034313 | KT033997 | KU612479 | KT034207 |
| 8 | *S. lissolab1atus* | Biyu River, Lanping, Yunnan | 99.153 | 26.980 |  | ihb201306134 |  | KT034320 | KT033996 |  | KT034205 |
| 8 | *S. lissolab1atus* | Biyu River, Lanping, Yunnan | 99.153 | 26.980 |  | ihb201306135 |  | KT034310 | KT033983 | KU612470 | KU612511 |
| 8 | *S. lissolab1atus* | Biyu River, Lanping, Yunnan | 99.153 | 26.980 |  | ihb201306139 |  | KT034315 | KT033995 | KU612481 |  |
| 8 | *S. lissolab1atus* | Biyu River, Lanping, Yunnan | 99.153 | 26.980 |  | ihb201306140 |  | KT034296 | KT033953 | KU612459 | KU612499 |
| 8 | *S.lantsangensis* | Biyu River, Lanping, Yunnan | 99.153 | 26.980 |  | ihb201306117 |  | KT034254 | KT033917 |  | KT034188 |
| 8 | *S.lantsangensis* | Biyu River, Lanping, Yunnan | 99.153 | 26.980 |  | ihb201306118 |  | KT034243 | KT033920 |  | KU612483 |
| 8 | *S.lantsangensis* | Biyu River, Lanping, Yunnan | 99.153 | 26.980 |  | ihb201306120 |  | KT034255 | KT033925 | KT034098 | KT034182 |
| 8 | *S.lantsangensis* | Biyu River, Lanping, Yunnan | 99.153 | 26.980 |  | ihb201306121 |  | KT034241 | KT033922 | KU612448 | KU612489 |
| 8 | *S.lantsangensis* | Biyu River, Lanping, Yunnan | 99.153 | 26.980 |  | ihb201306136 |  | KT034245 | KT033921 |  | KU612487 |
| 8 | *S.lantsangensis* | Biyu River, Lanping, Yunnan | 99.153 | 26.980 |  | ihb201306137 |  | KT034258 | KT033924 | KT034093 | KT034177 |
| 8 | *S.lantsangensis* | Biyu River, Lanping, Yunnan | 99.153 | 26.980 |  | ihb201306138 |  | KT034253 | KT033927 | KU612446 | KU612485 |
| 8 | *S. yunnanensis* | Biyu River, Lanping, Yunnan | 99.153 | 26.980 |  | ihb201306129 |  | KT034400 | KT034076 | KT034103 | KT034187 |
| 8 | *S. yunnanensis* | Biyu River, Lanping, Yunnan | 99.153 | 26.980 |  | ihb201306119 |  | KT034404 | KT034079 | KT034101 | KT034185 |
| 8 | *S. yunnanensis* | Biyu River, Lanping, Yunnan | 99.153 | 26.980 |  | ihb201306130 |  | KT034402 | KT034081 |  | KT034200 |
| 8 | *S. yunnanensis* | Biyu River, Lanping, Yunnan | 99.153 | 26.980 |  | ihb201306131 |  | KT034397 | KT034077 | KT034150 | KT034226 |
| 9 | *S. lissolab1atus* | Deqing River, Lanping, Yunnan | 99.176 | 26.928 |  | ihb201306108 |  | KT034284 | KT033987 | KU612455 | KU612496 |
| 9 | *S. lissolab1atus* | Deqing River, Lanping, Yunnan | 99.176 | 26.928 |  | ihb201306112 |  | KT034302 | KT033967 |  | KU612500 |
| 9 | *S. lissolab1atus* | Deqing River, Lanping, Yunnan | 99.176 | 26.928 |  | ihb201306113 |  | KT034289 | KT033986 | KU612463 | KU612505 |
| 9 | *S. lissolab1atus* | Deqing River, Lanping, Yunnan | 99.176 | 26.928 |  | ihb201306114 |  | KT034280 | KT033952 |  |  |
| 9 | *S. lissolab1atus* | Deqing River, Lanping, Yunnan | 99.176 | 26.928 |  | ihb201306115 |  | KT034321 | KT033992 |  | KT034208 |
| 9 | *S. lissolab1atus* | Deqing River, Lanping, Yunnan | 99.176 | 26.928 |  | ihb201306116 |  | KT034293 | KT033964 | KU612467 | KU612508 |
| 9 | *S.lantsangensis* | Deqing River, Lanping, Yunnan | 99.176 | 26.928 |  | ihb201306106 |  | KT034246 | KT033934 | KT034094 | KT034178 |
| 10 | *S. lissolab1atus* | Yingpan, Lanping, Yunnan | 99.145 | 26.480 |  | ihb201306080 |  | KT034282 | KT033959 | KU612456 |  |
| 10 | *S. lissolab1atus* | Yingpan, Lanping, Yunnan | 99.145 | 26.480 |  | ihb201306082 |  | KT034314 | KT033993 | KT034130 | KT034212 |
| 10 | *S. lissolab1atus* | Yingpan, Lanping, Yunnan | 99.145 | 26.480 |  | ihb201306096 |  | KT034307 | KT033978 |  | KU612501 |
| 10 | *S. lissolab1atus* | Yingpan, Lanping, Yunnan | 99.145 | 26.480 |  | ihb201306097 |  | KT034281 | KT033970 | KU612472 | KU612513 |
| 10 | *S. lissolab1atus* | Yingpan, Lanping, Yunnan | 99.145 | 26.480 |  | ihb201306100 |  | KT034316 | KT033994 | KU612480 | KT034209 |
| 10 | *S.lantsangensis* | Yingpan, Lanping, Yunnan | 99.145 | 26.480 |  | ihb201306086 |  | KT034257 | KT033923 |  | KU612486 |
| 10 | *S.lantsangensis* | Yingpan, Lanping, Yunnan | 99.145 | 26.480 |  | ihb201306087 |  | KT034244 | KT033929 | KU612449 | KU612490 |
| 10 | *S.lantsangensis* | Yingpan, Lanping, Yunnan | 99.145 | 26.480 |  | ihb201306089 |  | KT034249 | KT033931 | KT034097 | KT034181 |
| 10 | *S.lantsangensis* | Yingpan, Lanping, Yunnan | 99.145 | 26.480 |  | ihb201306090 |  | KT034252 | KT033933 |  |  |
| 10 | *S.lantsangensis* | Yingpan, Lanping, Yunnan | 99.145 | 26.480 |  | ihb201306091 |  | KT034242 | KT033919 |  |  |
| 10 | *S.lantsangensis* | Yingpan, Lanping, Yunnan | 99.145 | 26.480 |  | ihb201306092 |  | KT034251 | KT033918 | KT034099 | KT034183 |
| 10 | *S.lantsangensis* | Yingpan, Lanping, Yunnan | 99.145 | 26.480 |  | ihb201306093 |  | KT034256 | KT033926 | KT034100 | KT034184 |
| 10 | *S.lantsangensis* | Yingpan, Lanping, Yunnan | 99.145 | 26.480 |  | ihb201306095 |  | KT034250 | KT033930 | KU612447 | KU612488 |
| 10 | *S.lantsangensis* | Yingpan, Lanping, Yunnan | 99.145 | 26.480 |  | ihb201306088 |  | KT034240 | KT033915 |  | KT034206 |
| 10 | *S. yunnanensis* | Yingpan, Lanping, Yunnan | 99.145 | 26.480 |  | ihb201306101 |  | KT034405 | KT034082 | KT034092 | KT034176 |
| 10 | *S. yunnanensis* | Yingpan, Lanping, Yunnan | 99.145 | 26.480 |  | ihb201306083 |  | KT034396 | KT034073 | KT034152 | KT034228 |
| 10 | *S. yunnanensis* | Yingpan, Lanping, Yunnan | 99.145 | 26.480 |  | ihb201306084 |  | KT034399 | KT034078 | KT034102 | KT034186 |
| 10 | *S. yunnanensis* | Yingpan, Lanping, Yunnan | 99.145 | 26.480 |  | ihb201306085 |  | KT034398 | KT034074 |  | KU612520 |
| 10 | *S. yunnanensis* | Yingpan, Lanping, Yunnan | 99.145 | 26.480 |  | ihb201306094 |  | KT034403 | KT034080 | KU612482 | KU612521 |
| 11 | *S. nudiventris* | Jiuzhou, Yunlong, Yunnan |  |  |  |  | 2 | EU158027* |  |  |  |
| 12 | *S. lissolab1atus* | Jiezi, Yunlong, Yunnan |  |  |  |  | 2 | EU158046* |  |  |  |
| 12 | *S. lissolab1atus* | Jiezi, Yunlong, Yunnan | 99.325 | 25.595 |  | ihb201306098 |  | KT034317 | KT033989 | KT034124 | KT034203 |
| 12 | *S. lissolab1atus* | Jiezi, Yunlong, Yunnan | 99.325 | 25.595 |  | ihb201306099 |  | KT034318 | KT033990 |  |  |
| 13 | *S. lissolab1atus* | Yongping, Yunnan |  |  |  |  | 1 | AY954251* |  |  |  |
| 13 | *S. nudiventris* | Yongping, Yunnan |  |  |  |  | 1 | AY954253* |  |  |  |
| 13 | *S. yunnanensis* | Yongping, Yunnan |  |  |  |  | 1 | AY954252* |  |  |  |
| 14 | *S. lissolab1atus* | Baoshan, Yunnan |  |  |  |  | 1 | EU158052* |  |  |  |
| 15 | *S. lissolab1atus* | Erhai, Dali, Yunnan | 100.201 | 25.812 |  | SLR-1 |  | KT034276 | KT033954 | KT034123 | KT034202 |
| 15 | *S. lissolab1atus* | Erhai, Dali, Yunnan | 100.201 | 25.812 |  | SLR-2 |  | KT034277 | KT033955 |  |  |
| 15 | *S. lissolab1atus* | Erhai, Dali, Yunnan | 100.201 | 25.812 |  | SLR-3 |  | KT034278 | KT033956 |  |  |
| 15 | *S. yunnanensis* | Erhai, Dali, Yunnan | 100.201 | 25.812 |  | SYR-1 |  | KT034393 | KT034070 |  |  |
| 15 | *S. yunnanensis* | Erhai, Dali, Yunnan | 100.201 | 25.812 |  | SYR-2 |  | KT034394 | KT034071 |  |  |
| 15 | *S. yunnanensis* | Erhai, Dali, Yunnan | 100.201 | 25.812 |  | SYR-3 |  | KT034395 | KT034072 | KT034151 | KT034227 |
|  |  |  |  |  |  |  |  |  |  |  |  |
| Outgroup |  |  |  |  |  |  |  |  |  |  |  |
| *Cytb* | *S. malacanthus* | Longchuan River (Irrawaddy River) |  |  |  |  |  | AY954277 |  |  |  |
|  | *S. meridionalis* | Longchuan River (Irrawaddy River) |  |  |  |  |  | AY954285 |  |  |  |
|  | *S. meridionalis* | Longchuan River (Irrawaddy River) |  |  |  |  |  | AY954282 |  |  |  |
|  | *S. paoshanensis* | Longchuan River (Irrawaddy River) |  |  |  |  |  | AY954286 |  |  |  |
|  | *S. chongi* | Jinsha River (upper Yangtze River) |  |  |  |  |  | DQ126118 |  |  |  |
|  | *S. davidi* | Jinsha River (upper Yangtze River) |  |  |  |  |  | AY954257 |  |  |  |
|  | *S. dolichonema* | Jinsha River (upper Yangtze River) |  |  |  |  |  | DQ126116 |  |  |  |
|  | *S. griseus* | Jinsha River (upper Yangtze River) |  |  |  |  |  | EU158034 |  |  |  |
|  | *S. kozlovi* | Jinsha River (upper Yangtze River) |  |  |  |  |  | AY954256 |  |  |  |
|  | *S. prenanti* | Jinsha River (upper Yangtze River) |  |  |  |  |  | DQ126119  AY463521  EU158050  DQ058279  DQ058260 | |  |  |
|  | *S. wangchiachii* | Jinsha River (upper Yangtze River) |  |  |  |  |  |  |  |
|  | *S. lissolabiatus* | Red River |  |  |  |  |  |  |  |
|  | *G. eckloni* | Yellow River |  |  |  |  |  |  |  |
|  | *G. eckloni* | Yellow River |  |  |  |  |  |  |  |
|  | *G. przewalskii* | Yellow River |  |  |  |  |  | DQ058240 | |  |  |
|  | *G. przewalskii* | Yellow River |  |  |  |  |  | DQ058216 | |  |  |
|  | *B. callensis* |  |  |  |  |  |  | AF045974 |  |  |  |
|  | *B. guiraonis* |  |  |  |  |  |  | AF045972 |  |  |  |
|  | *B. sclateri* |  |  |  |  |  |  | AF045970 |  |  |  |
|  |  |  |  |  |  |  |  |  | |  |  |
| *CCR* |  |  |  |  |  |  |  |  | |  |  |
|  | *S. biddulphi* | Tarim River basin |  |  |  |  |  | JQ844133 | |  |  |
|  | *S. esocinus* | India |  |  |  |  |  | NC_022867 | |  |  |
|  | *S. macropogon* | Tsangpo River |  |  |  |  |  | NC_020339 | |  |  |
|  | *S. oconnori* | Tsangpo River |  |  |  |  |  | NC_020781 | |  |  |
|  | *S. richardsonii* | India |  |  |  |  |  | KC790369 | |  |  |
|  | *S. waltoni* | Tsangpo River |  |  |  |  |  | KC513574 | |  |  |

*Sequences were obtained from the four documents.

1. He D, Chen Y (2006) Biogeography and molecular phylogeny of the genus *Schizothorax* (Teleostei: Cyprinidae) in China inferred from cytochrome b sequences. Journal of Biogeography 33, 1448-1460.

2. Qi D, Chao Y, Guo S, et al. (2012) Convergent, parallel and correlated evolution of trophic morphologies in the subfamily schizothoracinae from the Qinghai-Tibetan plateau. PLoS One 7, e34070.

3. Yang J, Yang JX, Chen XY (2012) A re-examination of the molecular phylogeny and biogeography of the genus *Schizothorax* (Teleostei: Cyprinidae) through enhanced sampling, with emphasis on the species in the

Yunnan-Guizhou Plateau, China. J Zool Syst Evol Res 50, 184-191.
