# Supplementary material for: Genetic diversity and evolutionary history of the Schizothorax species complex in the Lancang River (upper Mekong)
Source: Ecol Evol. 2016 Jul 22;6(17):6023–36. doi: 10.1002/ece3.2319 (PMC5016629; doi:10.1002/ece3.2319)
Supplement: Supplementary file 4 — Table S2. Primers used for PCR and sequencing. [file ECE3-6-6023-s004.docx]

|  | Primer name | Primer sequence | Annealing  temperature (℃) | Cited sources |
| --- | --- | --- | --- | --- |
| *Cytb* | L14724 | GACTTGAAA AACCACCGTTG | 58-64 | [[1](#_ENREF_1)] |
|  | H15915 | CTCCGATCTCCGGATTACAAGAC |  |  |
| *CR* | GEDL200 | CACCCCTGGCTCCCAAAGCCAG | 58-64 | [[2](#_ENREF_2)] |
|  | GEDH860 | AGGGGTTTGACAAGAATAACAGGA |  |  |
| *RAG-1* | R1 2533F | CTGAGCTGCAGTCAGTACCATAAGATGT | 53 | [[3](#_ENREF_3)] |
|  | R1 4090R | CTGAGTCCTTGTGAGCTTCCATRAAYTT |  |  |
| *RAG-2* | RAG2-f2a | AARCGCTCMTGTCCMACTGG | 55 | [[4](#_ENREF_4)] |
|  | RAG2-R6a | TGRTCCARGCAGAAGTACTTG |  |  |

1. Xiao W, Zhang Y, Liu H: **Molecular systematics of Xenocyprinae (teleostei: cyprinidae): taxonomy, biogeography, and coevolution of a special group restricted in East Asia**. *Molecular phylogenetics and evolution* 2001, **18**(2):163-173.

2. Zhao K, Duan ZY, Peng ZG, Guo SC, Li JB, He SP, Zhao XQ: **The youngest split in sympatric schizothoracine fish (Cyprinidae) is shaped by ecological adaptations in a Tibetan Plateau glacier lake**. *Molecular ecology* 2009, **18**(17):3616-3628.

3. Lopez JA, Chen WJ, Orti G: **Esociform phylogeny**. *Copeia* 2004(3):449-464.

4. Lovejoy NR, Collette BB: **Phylogenetic relationships of new world needlefishes (Teleostei : Belonidae) and the biogeography of transitions between marine and freshwater habitats**. *Copeia* 2001(2):324-338.
